# Smallpox vaccination in a mouse model

**DOI:** 10.18699/VJGB-23-82

**Published:** 2023-10

**Authors:** S.N. Shchelkunov, A.A. Sergeev, S.A. Pyankov, K.A. Titova, S.N. Yakubitskiy

**Affiliations:** State Research Center of Virology and Biotechnology “Vector”, Rospotrebnadzor, Koltsovo, Novosibirsk region, Russia Institute of Cytology and Genetics of the Siberian Branch of the Russian Academy of Sciences, Novosibirsk, Russia; State Research Center of Virology and Biotechnology “Vector”, Rospotrebnadzor, Koltsovo, Novosibirsk region, Russia; State Research Center of Virology and Biotechnology “Vector”, Rospotrebnadzor, Koltsovo, Novosibirsk region, Russia; State Research Center of Virology and Biotechnology “Vector”, Rospotrebnadzor, Koltsovo, Novosibirsk region, Russia; State Research Center of Virology and Biotechnology “Vector”, Rospotrebnadzor, Koltsovo, Novosibirsk region, Russia

**Keywords:** smallpox, monkeypox, vaccinia virus, vaccination, intradermal injection, skin scarification, оспа, оспа обезьян, вирус осповакцины, вакцинация, внутрикожная инъекция, скарификация кожи

## Abstract

The monkeypox epidemic, which became unusually widespread among humans in 2022, has brought awareness about the necessity of smallpox vaccination of patients in the risk groups. The modern smallpox vaccine variants are introduced either intramuscularly or by skin scarification. Intramuscular vaccination cannot elicit an active immune response, since tissues at the vaccination site are immunologically poor. Skin has evolved into an immunologically important organ in mammals; therefore, intradermal delivery of a vaccine can ensure reliable protective immunity. Historically, vaccine inoculation into scarified skin (the s.s. route) was the first immunization method. However, it does not allow accurate vaccine dosing, and high-dose vaccines need to be used to successfully complete this procedure. Intradermal (i.d.) vaccine injection, especially low-dose one, can be an alternative to the s.s. route. This study aimed to compare the s.s. and i.d. smallpox immunization routes in a mouse model when using prototypic second- and fourth-generation low-dose vaccines (104 pfu). Experiments were conducted using BALB/c mice; the LIVP or LIVP-GFP strains of the vaccinia virus (VACV) were administered into the tail skin via the s.s. or i.d. routes. After vaccination (7, 14, 21, 28, 42, and 56 days post inoculation (dpi)), blood samples were collected from the retro-orbital venous sinus; titers of VACV-specific IgM and IgG in the resulting sera were determined by ELISA. Both VACV strains caused more profound antibody production when injected via the i.d. route compared to s.s. inoculation. In order to assess the level of the elicited protective immunity, mice were intranasally infected with a highly lethal dose of the cowpox virus on 62 dpi. The results demonstrated that i.d. injection ensures a stronger protective immunity in mice compared to s.s. inoculation for both VACV variants.

## Abstract (ru)

Необычно широко распространившаяся в 2022 г. эпидемия оспы обезьян среди людей привела к заключению о необходимости противооспенной вакцинации пациентов из групп риска. При этом современные варианты противооспенной вакцины вводят либо внутримышечно, либо скарификацией кожи. Внутримышечное введение не обеспечивает активного иммунного ответа, так как ткани, в которые при этом вводится вакцина, являются иммунологически бедными. Кожа эволюционно развилась в иммунологически важный орган млекопитающих, поэтому введение вакцины в дерму кожи может обеспечивать надежный протективный иммунный ответ. Исторически первым способом иммунизации стал метод инокуляции вакцины в скарифицированную кожу (с/к). Однако этот метод не обеспечивает точного дозирования вакцины, для успешного выполнения процедуры нужно использовать вакцину в высокой концентрации. Альтернативой методу с/к может служить процедура внутрикожной (в/к) инъекции вакцины, особенно при использовании ее в низкой концентрации. Целью настоящей работы было сравнение способов внутрикожной противооспенной иммунизации на модели мышей с применением прототипных вакцин второго и четвертого поколений в низкой дозе 104 БОЕ. Эксперименты выполняли на мышах линии BALB/c, штаммы LIVP или LIVP-GFP вируса осповакцины (VACV) вводили в кожу хвоста с/к или в/к способами. Через 7, 14, 21, 28, 42 и 56 дней после вакцинации (дпв) у мышей проводили забор проб крови из ретроорбитального венозного синуса и получали сыворотки, в которых методом ИФА определяли титры VACV-специфичных IgM и IgG. Оба штамма VACV обусловливали более выраженную продукцию антител при в/к инъекции по сравнению со с/к инокуляцией. Для проверки уровня развившегося протективного иммунитета на 62-й дпв мышей интраназально инфицировали высоколетальной дозой вируса оспы коров. Полученные результаты показали, что в/к инъекция обеспечивает развитие протективного иммунитета у мышей в значительно большей степени, по сравнению с с/к инокуляцией обоих вариантов VACV.

## Introduction

Smallpox (lat. variola) is an especially dangerous infectious disease that has claimed lives of many hundreds of millions of people over the past centuries. During smallpox epidemics, the death toll among the infected people could be as high as 30–40 %. The variola virus (VARV) is an infectious agent of this disease (Fenner et al., 1998).

The VARV was mostly transmitted amongst humans via the airborne or aerosol route during close personal contacts. The incubation period lasting one or two weeks was followed by abrupt onset of fever, headache, and sacral pain. Several days later, rash lesions appeared on the tongue as well as oral and oropharyngeal mucosa; maculopapular rash then developed on the face and hands, subsequently spreading over the entire body and progressing to pustules. By day 10–13 of illness, the pustules reached their maximum size, and then gradually flattened, dried, and evolved into scabs. By day 30–40 of the disease, the scabs fell off to leave reddish spots. These scabs subsequently left typical deep scars known as pockmarks in some body areas, mostly on the face (the pock-pitted face). Hence, smallpox survivors could be easily phenotypically differentiated from people who had not had this disease (Shchelkunov et al., 2005).

It turned out that smallpox survivors were not susceptible to it during the later epidemics. Many centuries ago, this fact apparently gave an idea to Indian and Chinese doctors to develop a procedure that subsequently became known as variolation (variola inoculation). According to this method, the infectious material obtained by rubbing scabs taken from epidemic patients was placed (inoculated) into skin incisions. People infected intradermally typically had a milder form of smallpox compared to the naturally occurring smallpox. After the infectious process, a characteristic scar was formed at the site of VARV inoculation into the skin. This procedure made people resistant to smallpox. However, 0.5–2.0 % of variolated individuals died, so this smallpox protection method has not become common (Fenner et al., 1988).

In the XVIII century, a smallpox-like disease in cattle and horses, which became known as cowpox, was reported in England. This disease was clinically characterized by development of skin rashes on animal bodies, most frequently on the udder and teats. The skin elements underwent typical evolutionary transformation stages (papules to vesicles to pustules); scabs and ulcers were subsequently formed. This infection was easily transmitted to people who had contacted the infected animals. In most cases, cowpox in humans had a mild course and was characterized by isolated topical lesions, mostly on hands and forearms, at skin microtrauma sites. After infection resolution, cicatrices resembling variolation scars were formed at former skin lesion sites. Furthermore, people who recovered from cowpox did not get infected during smallpox epidemics

Having gained this knowledge, an English physician Ed. Jenner inferred that people can be protected against smallpox by being preliminarily infected with cowpox. Starting with 1796, he conducted several experimental inoculations of the infectious material collected from pustules of cowpoxinfected humans into skin incisions (the skin scarification route) in people and, after some time, infected them with smallpox using the variolation procedure. In all the cases, people infected with the cowpox were resistant to smallpox infection. Ed. Jenner called the developed smallpox protection procedure “vaccination” (or vaccine inoculation, the term derived from Latin vacca – cow) (Fenner et al., 1988; Esparza et al., 2017).

It is noteworthy that it was not until one century after the invention of the smallpox vaccination method that the kingdom of viruses was discovered. However, it has only recently been found that different vaccinia virus (VACV) strains that have been used for immunization for a long time are closest to the horsepox virus rather than the cowpox virus in terms of their genomic organization (Tulman et al., 2006; Esparza et al., 2017).

Smallpox was completely eradicated by 1977 using mass smallpox vaccination and strict epidemiologic surveillance under the World Health Organization’s Global Smallpox Eradication Program (Fenner et al., 1988).

In the overwhelming majority of cases, the VACV was inoculated for smallpox vaccination by the skin scarification (s.s.) route. This procedure is relatively easy to perform but does not allow accurate dosing of the vaccine preparation; therefore, high-dose viral preparation needs to be used to ensure reliable immunization (Fenner et al., 1988; Jacobs et al., 2009; Sanchez-Sampedro et al., 2015).

Intradermal (i.d.) injection of the vaccine preparation can be a modern alternative to the s.s. route. This approach allows accurate vaccine dosing and ensures higher immunization reliability, so the dose of the administered vaccine can be reduced, which is especially important in the case of mass vaccination.

This study aimed to compare the effectiveness of i.d. and s.s. smallpox vaccination with low-dose VACV in a model of BALB/c mice. For correct comparison, the VACV was introduced by both routes within the same region of mouse tail skin for both procedures. The clonal variant of the LIVP strain and the constructed recombinant LIVP-GFP (mutant with respect to viral thymidine kinase), which can be regarded as prototypic second- and fourth-generation smallpox vaccines, respectively, were used as study objects.

## Materials and methods

Viruses and cell culture. Clonal variant 14 of the VACV LIVP strain produced by limiting dilution and triple plaque purification using agarose overlay (Yakubitskiy et al., 2015), the mutant LIVP-GFP, with inactivated virus thymidine kinase gene, generated based on it (Petrov et al., 2013), and the cowpox virus (CPXV) strain GRI-90 (Shchelkunov et al., 1998) were used in this study. The viruses were grown and titrated using the African green monkey kidney cells line CV-1 from the collection of the State Research Center of Virology and Biotechnology “Vector”, Federal Service for Surveillance on Consumer Rights Protection and Human Wellbeing

Animals. BALB/c mice were procured from the Laboratory Animals Farm of the SRC VB Vector. The experimental animals were fed a standard diet with a sufficient amount of water, in compliance with the veterinary laws and the requirements for the humane care and use of laboratory animals. Animal manipulations were approved by the Bioethics Committee of SRC VB Vector (Protocol No. 02-06.2022 dated June 28, 2022).

Pathogenicity assessment of VACV strains. Three-weekold BALB/c mice weighing 10–12 g (10 animals per group) were used in the studies to assess the pathogenicity of the VACV LIVP and LIVP-GFP strains upon intranasal (i.n.) infection. Mice preliminarily subjected to inhaled anesthesia with diethyl ester received 50 μL of virus-containing fluid at a dose of 107 plaque-forming units (pfu) or normal saline inoculated into the nasal cavity. The animals were followed up for 14 days; clinical signs of infection and animal deaths were documented.

The score grading system for assessing the revealed symptoms was as follows: 0 – no signs of the disease; 1 – slightly ruffled hair coat; 2 – significantly ruffled hair coat; 3 – significantly ruffled hair coat and hunched posture or conjunctivitis; 4 – labored breathing or remaining immobile; and 5 – death.

Each mouse was weighed every two days. The arithmetic mean mouse body weight in each group at each time point was calculated and expressed as a percentage from the baseline value. Data diffusion with respect to the mean value was presented as standard deviation of the mean and also expressed as a percentage.

Immunization of mice. Female BALB/c mice starting with age of 6–7 weeks (body weight, 16–19 g) were immunized by intradermal (i.d.) injection or skin scarification (s.s.) using VACV LIVP or LIVP-GFP at a dose of 104 pfu.

When performing an i.d. injection or s.s. inoculation, the inoculation site (the dorsal side of the tail, approximately 1 cm from its base) was pretreated with 70 % ethanol. In the case of i.d. injection, 20 μL of viral material (104 pfu) or normal saline (control group) was inoculated according to the procedure described earlier (Shchelkunov et al., 2022a). For s.s. immunization, 10 skin incisions were made using a 26G needle (0.45 × 16 mm) within the uppermost layer of epidermis. Viral material (104 pfu) or normal saline (control group) (5 μL) was immediately applied onto the damaged skin and allowed to be absorbed into the skin.

On days 7, 14, 21, 28, 42, and 56 post inoculation (dpi) with the LIVP or LIVP-GFP viruses, blood samples were collected from the retro-orbital venous sinus in mice (six animals from each group) according to the procedure described previously (Shchelkunov et al., 2022a).

Serum preparations were obtained from individual blood samples of mice by centrifuging blood cells. Serum samples obtained from mouse blood were stored at –20 °С.

Enzyme-linked immunosorbent assay of serum samples. Enzyme-linked immunosorbent assay of individual serum samples of mice was carried out according to the procedure described previously (Shchelkunov et al., 2020). Purified VACV LIVP preparation was used as an antigen. The geometric means of log reciprocal titer of VACV-specific IgM and IgG were determined for the study groups, and the confidence intervals were calculated for the 95 % matching between each sample and the total population.

Assessment of protectivity in immunized mice. On 62 dpi, the groups of animals immunized with the LIVP or LIVP-GFP viruses and control animals were i.n. inoculated with CPXV GRI-90 at a dose of 46 LD50 (9.4 × 105 pfu/mouse). The animals were followed up for 14 days, and their deaths were documented

The data were obtained for groups consisting of six animals immunized, either i.d. or by s.s., with VACV LIVP or LIVP-GFP, as well as groups of non-immunized mice and non-infected animals (the negative control) or animals infected with CPXV GRI-90 (the positive control).

Statistical analysis. Statistical analysis and comparison of the results was carried out with standard methods using the Statistica 13.0 software package (StatSoft Inc., 1984–2001). The 50 % lethal dose (LD50) was calculated using the Spearman– Karber method according to the number of animals that had died (Sachs, 1972). The p-value < 0.05 was considered statistically significant.

## Results

Comparison of the pathogenic properties of LIVP and LIVP-GFP strains intranasally inoculated to mice

**Fig. 1.**
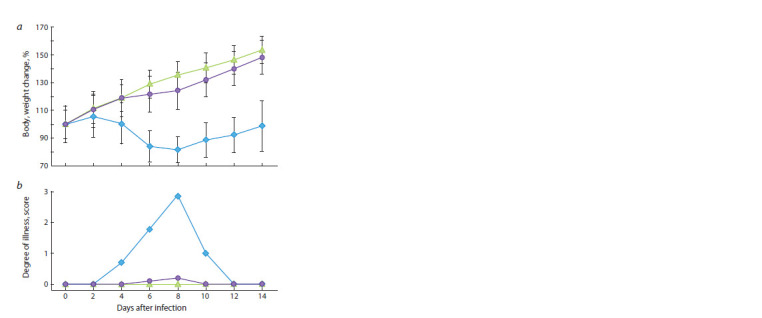
Changes in mouse body weight (a) and clinical manifestations of infection (b) after intranasal inoculation of the LIVP (shown in blue) or LIVP-GFP (shown in violet) viruses at a dose of 107 pfu. The mean data for groups consisting of 10 animals infected with the respective viruses and the control group (shown in green) are presented.

Three-week-old BALB/c mice were used to assess the pathogenicity of the VACV LIVP and LIVP-GFP strains in this study. The mice were i.n. inoculated with the viruses at a dose of 107 pfu. For VACV LIVP, pronounced clinical manifestations of the infection were observed starting with 4 dpi; their maximum intensity was detected on 8 dpi; and the animals recovered after 10 dpi (Fig. 1, b). The disease was accompanied by significant body weight loss of mice (see Fig. 1, a).

**Fig. 2.**
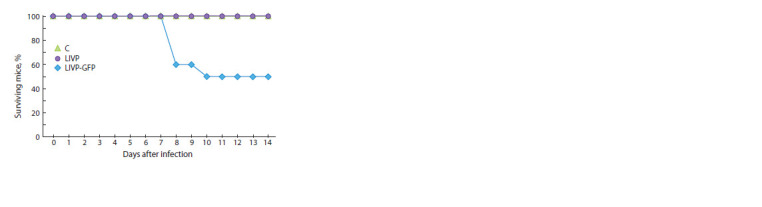
Deaths of mice intranasally inoculated with the LIVP (shown in blue) or LIVP-GFP (shown in violet) viruses at a dose of 107 pfu. The control group consisted of non-infected animals (shown in green).

Under the same conditions, the LIVP-GFP virus with the mutant thymidine kinase gene caused minimal clinical manifestations of infection 6–8 dpi (see Fig. 1, b) and an insignificant body weight loss in infected animals compared to those in the control group (see Fig. 1, a). I.n. inoculation of mice with the LIVP strain resulted in the death of 50 % animals, whereas all the animals survived the inoculation with the LIVP-GFP strain (Fig. 2).

The results demonstrate that VACV LIVP was significantly attenuated in the case of inactivation of the thymidine kinase gene that had occurred when producing the recombinant LIVP-GFP strain.

Comparison of changes in the development of humoral immune response to vaccination of mice with the LIVP and LIVP-GFP viruses over time

Adult BALB/c mice, starting with the age of 6–7 weeks, were vaccinated by i.d. injection or s.s. inoculation with low-dose VACV LIVP or LIVP-GFP (104 pfu).

**Fig. 3.**
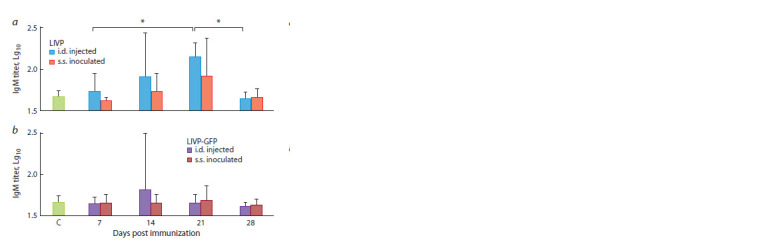
Titers of VACV-specific IgM in serum samples from mice immunized with the LIVP (a) or LIVP-GFP viruses (b). C – serum samples of mice that received normal saline (control group). * Statistically significant differences with p < 0.05.

Upon i.d. injection of the LIVP virus, significant production of VACV-specific IgM was observed as early as on 7 dpi; its maximum level was reached by 21 dpi, while the IgM titer dropped to the level observed for the negative control group by 28 dpi and later. Therefore, the results of testing IgM in mouse serum samples are shown in Fig. 3 only for the time points of 7, 14, 21, and 28 dpi. Immunization of mice with the LIVP virus by s.s. inoculation resulted in later and less marked IgM production (see Fig. 3, a).

Both in the case of i.d. and s.s. vaccination of mice with the LIVP-GFP virus (104 pfu), IgM production was minimal; there were no significant differences compared to the IgM level in the control serum samples of non-immunized animals (see Fig. 3, b).

**Fig. 4.**
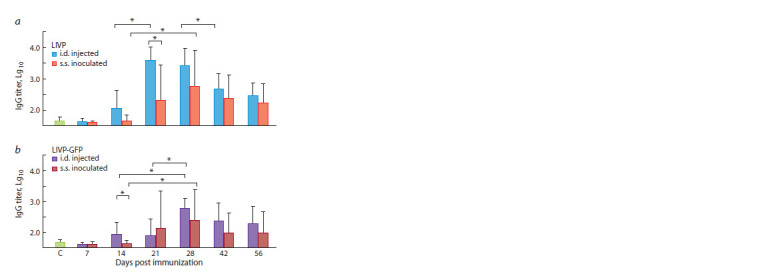
Titers of VACV-specific IgG in serum samples of mice immunized with the LIVP (a) or LIVP-GFP (b) viruses. C – serum samples of mice that received normal saline (control group). * Statistically significant differences with p < 0.05.

Much higher titers of VACV-specific immunoglobulins IgG were produced compared to those of IgM (see Figs. 3 and 4). After i.d. vaccination with the LIVP virus, significant IgG production was observed as early as on 7 dpi, reaching its maximum on 21 dpi and remaining at virtually the same level until 28 dpi. The titer of VACV-specific IgG then gradually decreased by 42 and 56 dpi (see Fig. 4, a). For s.s. inoculation of the LIVP virus to mice, synthesis of specific IgG was delayed and had lower intensity compared to i.d. immunization (see Fig. 4, a).

When mice were i.d. inoculated with the LIVP-GFP virus, the IgG production level was considerably lower compared to that observed after vaccination with LIVP; the production of these antibodies was maximal on 28 dpi (see Fig. 4, b). S.s. inoculation of the LIVP-GFP virus resulted in lowerintensity production of analyzed IgG (see Fig. 4).

Assessment of protection against the lethal orthopoxvirus infection in immunized mice

**Fig. 5.**
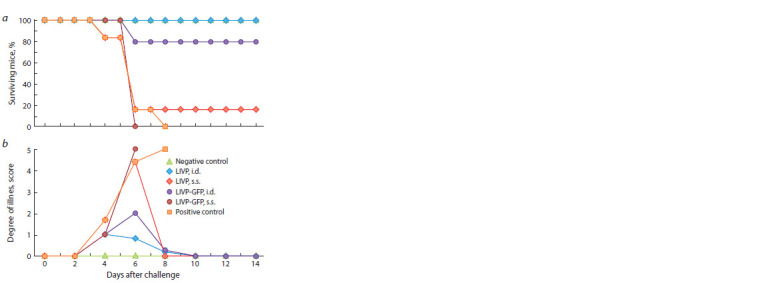
Deaths (a) and clinical manifestations of infection in mice (b) immunized with the LIVP or LIVP-GFP viruses (104 pfu) after being intranasally infected with CPXV GRI-90 at a dose of 46 LD50 on 62 dpi. The mean data for the groups consisting of six animals immunized with the respective viruses, as well as the non-immunized and non-infected mice (the negative control group) or mice infected with CPXV GRI-90 (the positive control group) are presented.

In order to assess how the VACV strains under study, as well as the i.d. and s.s. vaccine administration routes, affect the development of protective immunity against orthopoxvirus reinfection in mice, groups of mice immunized with the LIVP or LIVP-GFP, as well as control (non-immunized) animals, were i.n. inoculated with CPXV GRI-90 at a dose of 46 LD50 on 62 dpi. The results of these experiments (Fig. 5) demonstrate that only the group of mice i.d. immunized with the LIVP virus were fully protected. In the group of mice vaccinated with the same virus through the s.s. route, 83 % of animals died after being infected with CPXV-GRI (see Fig. 5, a).

I.d. injection of the LIVP-GFP virus protected 80 % of mice against reinfection with CPXV-GRI under the same conditions, while all the mice s.s. inoculated with LIVP-GFP died (see Fig. 5, a). The level of protection against the lethal CPXV infection in mice correlated with the intensity of clinical manifestations of this infection (see Fig. 5, b).

Hence, i.d. low-dose immunization with VACV (104 pfu) used in this study for mice is obviously more effective compared to s.s. inoculation in the development of protective immunity against heterologous orthopoxvirus infection (the cowpox virus).

## Discussion

The large-scale epidemic of monkeypox among humans that spread to all continents in 2022 (Harapan et al., 2022; Shchelkunova, Shchelkunov, 2023) has put the question about mass vaccination against this infection in the risk groups on the agenda. Important issues were the need to properly choose the type of vaccine and the optimal route of smallpox vaccine administration.

The first-generation live smallpox vaccine is a VACV preparation produced by viral replication in skin of calves or other animals. Recent studies have shown that these vaccines consist of a mixture of different VACV variants (Osborne et al., 2007; Qin et al., 2011).

In present-day conditions, the VACV vaccine strains obtained by isolating clonal variants from first-generation vaccines are produced on mammalian cell cultures, and these preparations are known to be second-generation smallpox vaccines (Sanchez-Sampedro et al., 2015). Application of firstand second-generation smallpox vaccines for mass vaccination is currently limited because of the relatively high risk of severe complications (Fenner et al., 1988; Sanchez-Sampedro et al., 2015), since the number of compromised people, including those infected with HIV, has recently increased.

Third-generation attenuated smallpox viruses (having reduced pathogenicity) are produced by multiple passages of a certain VACV strain in the cell culture of a heterologous host. This process is accompanied by emergence of VACV variants carrying spontaneous deletions and mutations in the viral genome (Jacobs et al., 2009; Olson, Shchelkunov, 2017; Albarnaz et al., 2018).

The novel approach to producing fourth-generation smallpox vaccines consists in introducing targeted deletions/ insertions that disrupt selected viral genes and lead to VACV attenuation by genetic engineering (Yakubitskiy et al., 2015; Li et al., 2017; Shchelkunov et al., 2022b).

First-generation live smallpox vaccine based on the VACV LIVP strain is used for smallpox immunization in Russia. The LIVP strain was produced by epicutaneous passaging of the Lister vaccine strain provided by the Lister Institute (Elstree, UK) in rabbits and calves. A preparation of this vaccine is the virus grown in scarified calf skin (Perekrest et al., 2013). The patients receive this vaccine via s.s. inoculation.

We used the LIVP strain to produce and characterize the clonal variant of LIVP (Yakubitskiy et al., 2015) that can be viewed as a prototype of second-generation smallpox vaccine. The recombinant LIVP-GFP strain with inactivated thymidine kinase gene generated based on it (Petrov et al., 2013) is a prototype variant of fourth-generation smallpox vaccine.

At the first stage of this study, we compared the pathogenicities of the LIVP and LIVP-GFP strains. The sensitivity of mice to orthopoxviruses significantly depends on their age (Shchelkunov et al., 2005); therefore, young (3-week-old) BALB/c mice were used in the experiments. The animals were i.n. inoculated with the viruses, since this route imitates the natural route of infection and ensures the highest sensitivity of mice to this infection (Hughes et al., 2020; Shchelkunov et al., 2021).

It turned out that after i.n. inoculation of young mice with the LIVP strain (107 pfu), it induced clinically apparent infection (see Fig. 1) resulting in death of 50 % of animals (see Fig. 2). Meanwhile, the LIVP-GFP strain led only to mild signs of the disease in mice (see Fig. 1) and complete recovery (see Fig. 2). Therefore, inactivation of the thymidine kinase gene in LIVP-GFP resulted in its substantial attenuation compared to the parental LIVP strain, which is consistent with the results obtained for other VACV strains (Taylor et al., 1991; Jacobs et al., 2009).

Numerous studies have previously demonstrated that s.s. immunization with second- and fourth-generation VACVbased vaccines at doses of at least 105–106 pfu fully protected mice against repeated lethal orthopoxvirus infection (Melamed et al., 2007; Jacobs et al., 2009; Shchelkunov et al., 2022a).

In this work, we studied the feasibility of reducing the dose of prototypic smallpox vaccines to 104 pfu when performing s.s. inoculation or i.d. injection to mice. For correct comparison, the VACV was introduced by the s.s. and i.d. routes within the same region of mouse tail skin.

Adult mice (aged 6–7 weeks) with a mature immune system were used for studying the immunogenicity of VACV LIVP and LIVP-GFP. The antibody response is known to make the most significant contribution to the development of adaptive immune response to VACV vaccination (Belyakov et al., 2003; Moss, 2011). Therefore, we studied changes in the synthesis of VACV-specific IgM and IgG after i.d. or s.s. vaccination of mice with the LIVP or LIVP-GFP strains. The results of these experiments demonstrated (see Figs. 3 and 4) that both VACV strains ensured more profound antibody production upon i.d. injection compared to s.s. inoculation. Meanwhile, statistically significant differences in the results between the compared groups were revealed only for IgG values on 21 dpi for LIVP (see Fig. 4, a) and 14 dpi for LIVP-GFP (see Fig. 4, b). No statistically significant differences in the results were observed for IgM (see Fig. 3).

In order to assess the level of protective immunity that developed in mice in response to s.s. or i.d. immunization with the LIVP or LIVP-GFP viruses, these animals were subjected to i.n. infection with a highly lethal dose of CPXV. It was considered to be the most adequate approach to assessing the effectiveness of VACV vaccination on the mouse model (Ferrier-Rembert et al., 2007; Melamed et al., 2007). The results (see Fig. 5) demonstrated that i.d. injection ensured a much stronger protective immunity compared to s.s. inoculation of the VACV. Only i.d. low-dose immunization with the LIVP strain fully protected mice against the lethal CPXV infection. The attenuated LIVP-GFP strain did not form a sufficiently strong protective immunity under the same conditions. S.s. inoculation with VACV LIVP or LIVP-GFP at the selected low dose did not protect animals against reinfection with CPXV (see Fig. 5).

## Conclusion

These findings give grounds for inferring that i.d. injection of both studied VACV variants induces a much stronger protective immunity in mice compared to s.s. inoculation of these viruses at the same dose. In addition to more accurate vaccine dosing for i.d. immunization compared to the s.s. route, the former one is associated with less significant skin damage, thus substantially reducing the intensity of inflammation reaction that impedes efficient VACV replication and lowering the risk of bacterial infection at the vaccination site (Shmeleva et al., 2022). When using an attenuated fourth-generation vaccine with reduced specific immunogenicity for smallpox immunization, a higher dose of VACV needs to be used as compared to that of the second-generation vaccine.

## Conflict of interest

The authors declare no conflict of interest.
